# Exposure to fine particulate matter (PM_2.5_) from non-tobacco sources in homes within high-income countries: a systematic review

**DOI:** 10.1007/s11869-022-01288-8

**Published:** 2022-11-28

**Authors:** Shuying Wei, Sean Semple

**Affiliations:** 1 Faculty of Health Sciences and Sport, University of Stirling, Stirling, FK9 4LA UK; 2 Institute for Social Marketing and Health, University of Stirling, Stirling, FK9 4LA UK

**Keywords:** PM_2.5_, Indoor air quality, Exposure assessment, High-income countries

## Abstract

The health impacts associated with exposure to elevated concentrations of fine particulate matter (PM_2.5_) are well recognised. There is a substantial number of studies characterising PM_2.5_ concentrations outdoors, as well as in homes within low- and middle-income countries. In high-income countries (HICs), there is a sizeable literature on indoor PM_2.5_ relating to smoking, but the evidence on exposure to PM_2.5_ generated from non-tobacco sources in homes is sparse. This is especially relevant as people living in HICs spend the majority of their time at home, and in the northern hemisphere households often have low air exchange rates for energy efficiency. This review identified 49 studies that described indoor PM_2.5_ concentrations generated from a variety of common household sources in real-life home settings in HICs. These included wood/solid fuel burning appliances, cooking, candles, incense, cleaning and humidifiers. The reported concentrations varied widely, both between sources and within groups of the same source. The burning of solid fuels was found to generate the highest indoor PM_2.5_ concentrations. On occasion, other sources were also reported to be responsible for high PM_2.5_ concentrations; however, this was only in a few select examples. This review also highlights the many inconsistencies in the ways data are collected and reported. The variable methods of measurement and reporting make comparison and interpretation of data difficult. There is a need for standardisation of methods and agreed contextual data to make household PM_2.5_ data more useful in epidemiological studies and aid comparison of the impact of different interventions and policies.

**Supplementary Information:**

The online version contains supplementary material available at 10.1007/s11869-022-01288-8.

## Introduction

Air pollution is a major hazard to public health globally, with nine out of ten people exposed to concentrations that exceed the World Health Organization (WHO) guidance limits. Poor outdoor air quality claims 4.2 million lives every year, and indoor air pollution accounts for 3.8 million annual deaths (World Health Organization 2021a). Particulate matter (PM) is one of the most common air pollutants that is associated with human health harms when it exceeds regulatory levels (Centers for Disease Control and Prevention 2021). PM_2.5_, PM less than 2.5 μm in diameter, is one of the most harmful pollutants to inhale due to its effects on health (Kelly and Fussell 2015; Schraufnagel et al. 2019).

The adverse health effects associated with exposure to PM_2.5_ are now well recognised in public health research. Studies have shown that exposure to elevated concentrations of PM_2.5_ is associated with an increased risk of hospitalisation for cardiopulmonary illnesses such as asthma, ischemic heart disease and cardiac failure (Du et al. 2016; Xing et al. 2016; Hayes et al. 2020). In addition to being linked to morbidity, chronic exposure to PM_2.5_ can also lead to a higher mortality risk for lung cancer and cardiovascular diseases (Arden Pope et al. 2011, 2020). The health effects of PM_2.5_ extend beyond the cardiopulmonary system. Recent studies have found associations between PM_2.5_ and the incidence of chronic kidney disease, type 2 diabetes and cerebrovascular disease (Li et al. 2017; Carey et al. 2018; Bowe et al. 2019; Ghazi et al. 2021). There is also emerging evidence to suggest that dementia, autism, depression and other mental health disorders may be related to long-term exposure (Lam et al. 2016; Braithwaite et al. 2019; Shi et al. 2020). Given the detrimental impact that PM_2.5_ has on health, there is a need to better understand how human exposure takes place. Characterising and investigating personal exposure to PM_2.5_ will help tackle emission sources and/or change behaviour to reduce exposure, which should, in turn, reduce the burden of air pollution-related illnesses.

PM_2.5_ is a ubiquitous pollutant coming from an array of emission sources. Although air pollution is most commonly associated with outdoor environments, PM_2.5_ generated from indoor sources and breathed in within the home setting is likely to make up a considerable proportion of total population inhaled dose. Even in the twenty-first century, 2.8 billion people still rely on burning solid fuels for heating, cooking and lighting (Bonjour et al. 2013). Indoor PM_2.5_ concentrations in low- and middle-income countries (LMICs) vary widely and are dependent on the type of combustion device and fuel used. Indoor concentrations of PM_2.5_ in LMICs often far exceed the WHO air quality guideline (AQG), which currently stand at 15 μg/m^3^ in 24 h and 5 μg/m^3^ annually (World Health Organization 2021b). For example, in homes with traditional solid fuel burning stoves in India (Arif and Parveen 2021), Mongolia (Lim et al. 2018) and Honduras (Young et al. 2019), mean 24-h indoor PM_2.5_ concentrations have been shown to exceed 200 μg/m^3^. Indoor air quality in LMICs has been extensively studied in recent decades owing to its associated adverse health impacts and implied socioeconomic inequalities. Investigation into indoor PM_2.5_ in LMICs continues, especially as interventions aimed at tackling the problem have had varied success (Budya and Yasir Arofat 2011; Hanna et al. 2012; Mortimer et al. 2017).

In contrast to LMICs, literature on indoor PM_2.5_ concentrations in high-income countries (HICs) is comparatively scarce despite it also being a relevant and substantial global problem. Some studies have characterised indoor PM_2.5_ concentrations in non-residential places within HICs, including offices (Jones et al. 2021), schools (Carrion-Matta et al. 2019), prisons (Semple et al. 2017), restaurants (El‐Sharkawy and Javed 2018) and other microenvironments. However, there are only a small number of studies that have characterised PM_2.5_ generated from sources in residential settings within HICs. It is important that the health impacts of household indoor PM_2.5_ levels in HICs are not overlooked, especially as people in HICs spend 90% of their time indoors, with almost 70% of that being at home (Klepeis et al. 2001; Delgado-Saborit et al. 2011), with even higher proportions of time spent at home during the COVID-19 pandemic (O’Donnell et al. 2021). By far, the most investigated source of PM_2.5_ within home settings in HICs is second-hand tobacco smoke. Studies consistently show that the concentration of indoor PM_2.5_ is significantly higher in smoking homes than non-smoking homes and often exceeds the WHO AQG (Semple et al. 2015; Zhang et al. 2020). The burning of solid or biomass fuels for the purpose of heating is one of the few non-tobacco household sources that has been investigated in HICs (Schluger 2014; Fleisch et al. 2020; Chakraborty et al. 2020). Other indoor PM_2.5_ sources have received little attention, despite their commonality within residential settings. These include cooking, cleaning and the combustion of material other than biomass fuel such as candles and incense.

The characterisation of PM_2.5_ in outdoor environments has been studied extensively in HICs. Databases have been compiled to show the longitudinal changes in outdoor PM_2.5_ concentrations, as well as indicating the real-time PM_2.5_ at local levels (Air Quality in Scotland 2022; Department for Environment Food & Rural Affairs 2022). There are also emerging citizen networks, such as PurpleAir that report both outdoor and indoor PM_2.5_ (PurpleAir 2022). Despite increasing awareness of the need to characterise indoor PM_2.5_, research into concentrations within home settings in HICs is relatively uncommon. In addition, most studies that report residential PM_2.5_ concentrations in HICs focus primarily on health outcomes (Habre et al. 2014; Karottki et al. 2014). It is often not obvious from the title of the articles that the studies involve measuring indoor PM_2.5_ thus making it difficult for those interested in the field to readily access or identify what has already been achieved. This systematic review, therefore, intends to identify, collate and appraise all relevant studies that investigate the indoor PM_2.5_ concentrations generated from common household sources in HICs and provide a comprehensive overview. The following research questions will be addressed in this systematic review:

1. What are the indoor concentrations of PM_2.5_ generated from common sources (excluding tobacco or e-cigarettes) in homes within HICs?
2. How do indoor concentrations of PM_2.5_ in homes within HICs compare to the WHO air quality guideline 2021?
3. What are the methods used in existing studies to measure and report concentrations of PM_2.5_ in homes within HICs?

By reviewing the current literature and drawing comparisons between various sources of PM_2.5_, this review aims to highlight the direction in which future research in the field should focus, and ultimately benefit the health of people living in HICs who are at risk of exposure to elevated concentrations of PM_2.5_ at home.

## Materials and methods

This systematic review was performed following the best practices outlined by the Centre for Reviews and Dissemination (Centre for Reviews and Dissemination 2009) and the Preferred Reporting Items for Systematic Reviews and Meta-Analyses (PRISMA) (Page et al. 2021).

### Search methods

A literature search was conducted using the PubMed database. The search strategy consisted of key terms covering three topic areas; air quality, emission source and setting. Search terms used to describe air quality included; indoor, home, residential, household, particulate matter and PM_2.5_. Exact names of household products or activities that generate indoor PM_2.5_ in home settings were used to search for emission source. Examples of these sources are woodstove, cooking fume, candle and humidifier. As for the setting, due to there being very few relevant studies conducted in HICs, the Boolean Logic “NOT” function was employed to exclude LMICs where studies concerning levels of indoor PM_2.5_ are most commonly conducted. Details of the search strings are provided in Supplementary Information 1. On the account of the envisaged scarcity of studies in the area of interest, there was no restriction on publication date and the search included all studies through to January 2022.

### Eligibility criteria

Studies were included if they met the following eligibility criteria: (1) conducted in HICs as defined by the World Bank in 2021 as having a gross national income per capita above 12,695 USD (The World Bank 2021); (2) PM_2.5_ concentrations measured and reported in µg/m^3^; (3) PM_2.5_ concentrations measured in real-life indoor residential settings (i.e. not laboratory settings, or home settings with highly controlled variables); and (4) the exposure to PM_2.5_ was objectively measured and was not a subjective assessment or self-reported proxy for exposure. Studies were excluded if they were not published in English, or reported PM_2.5_ concentrations generated from tobacco combustion (e.g. cigarette or pipe smoking) or e-cigarette sources (vaping). A post hoc decision was made during full-text screening stage about studies that sampled in both smoking and non-smoking homes; studies were excluded if the reported data could not be separated from smoking and non-smoking households.

### Selection process

The information from retrieved articles was imported into an Excel spreadsheet. After duplicates were removed, one researcher [SW] screened the titles and, where applicable, abstracts to identify relevant studies according to the eligibility criteria. Full-text articles were assessed if the relevance of a study was not obvious from its title or abstract. The second researcher [SS] randomly selected 10% of all retrieved articles and independently assessed the studies’ relevance to the research questions and whether they met the inclusion criteria. The random selection of the 10% sample was performed in R using Dplyr with the slice_sample function. The initial agreement on studies’ eligibility was 98% between the two researchers; discrepancies were resolved after discussion. Reference checking for additional relevant articles was carried out to maximise the capture of related studies; references were cited by the included studies as well as those citing the included studies.

### Data extraction

A data extraction form was designed and piloted before its application to all included studies. The extracted data was organised into two categories, one being study characteristics such as sample size, enrolment period and country where the study was conducted and the other category being methods of exposure assessment in which the following data were recorded: PM_2.5_ source, sampling duration, measurement device, location of measurement, type of measurement (static or personal) and main findings. The data extraction was completed by one researcher [SW] with the second [SS] cross-checking approximately 10% (*n* = 7) of studies to identify and minimise errors. The sampling of studies for cross-checking was conducted through selection of the 4th row and then every subsequent 10th row thereafter on the data extraction spreadsheet.

### Quality appraisal

The exposure assessment methods in included studies were appraised for their risk of bias. The appraisal was carried out using three criteria from the National Institutes of Health’s quality assessment tool for observational cohort and cross-sectional studies (National Institutes of Health 2021). The criteria were as follows:

1. For exposures that can vary in amount or level, did the study examine different levels of the exposure as related to the outcome (e.g., categories of exposure, or exposure measured as continuous variable)?
2. Were the exposure measures (independent variables) clearly defined, valid, reliable and implemented consistently across all study participants?
3. Was the exposure(s) assessed more than once over time?

Studies that answered “yes” to all three criteria were rated as low risk of bias, one “no” as being medium risk and two “no’s” as having high risk. All studies were included in data synthesis despite their levels of risk of bias.

## Results

### Study selection

**Fig. 1:**
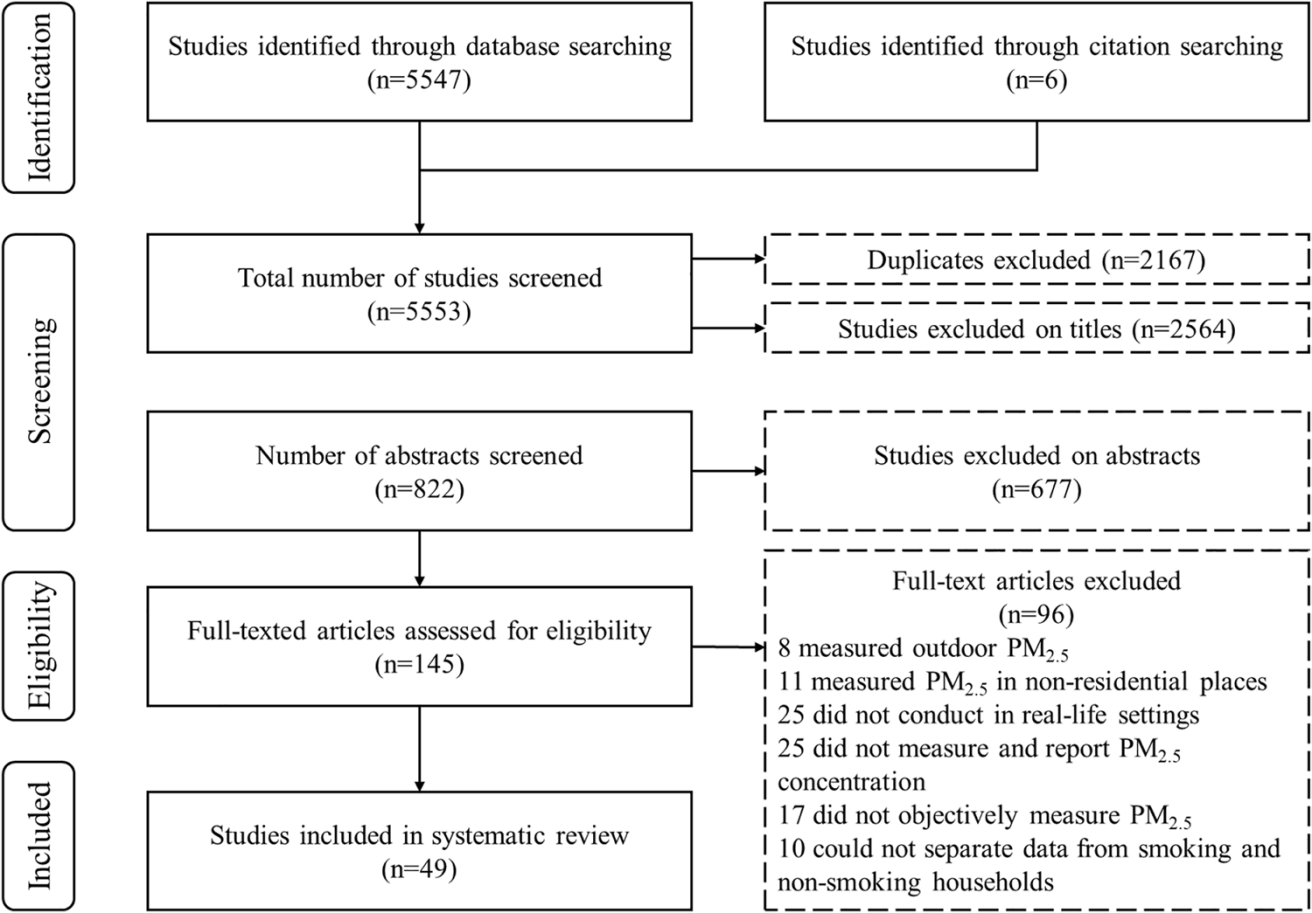
Flow diagram of the study selection process

A total of 5553 articles were retrieved from the literature search on PubMed and by reference-checking. After removing 2167 duplicates, 2564 studies were excluded based on their titles and a further 677 on their abstracts. The remaining 145 articles proceeded onto the full-text screening stage in which 96 were excluded due to the following reasons: did not measure and report PM_2.5_ concentrations; measured PM_2.5_ concentrations in outdoor, non-residential locations, laboratories or home settings with highly controlled variables; did not objectively measure exposure to PM_2.5_; and could not separate data from smoking and non-smoking homes. Thus, 49 studies were included in this systematic review (Fig. 1).

### Study characteristics

**Fig. 2:**
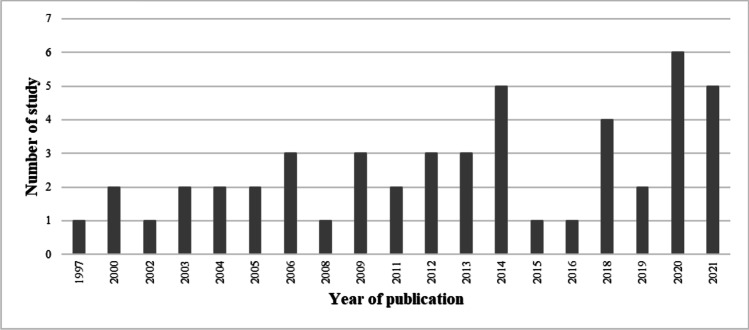
Number of included studies published in each year

Supplementary Information 2 details the main characteristics of included studies. There were 22 studies conducted in Europe; five in the UK (Wigzell et al. 2000; Nasir and Colbeck 2013; O’Leary et al. 2018; Chakraborty et al. 2020; Shehab et al. 2021); three in Portugal (Alves et al. 2020; Madureira et al. 2020; Marques and Pitarma 2020); two each in Sweden (Molnar et al. 2005; Omelekhina et al. 2022), Italy (Frasca et al. 2018; Pietrogrande et al. 2021) and Greece (Sarigiannis et al. 2014; Assimakopoulos et al. 2018); one each in Finland (Siponen et al. 2019), Norway (Wyss et al. 2016), Denmark (Karottki et al. 2014), Germany (Salthammer et al. 2014), Poland (Jedrychowski et al. 2006), Switzerland (Monn et al. 1997) and Belgium (Stranger et al. 2009); and one study was multicentric (UK and the Republic of Ireland) (Semple et al. 2012). There was only one study each from Asia (Japan) (Ohura et al. 2005), the Middle East (Kuwait) (Yassin et al. 2012), Oceania (Australia) (Mazaheri et al. 2018) and South America (Chile) (Rojas-Bracho et al. 2002). North America had the most studies, with 18 conducted in the USA (Abt et al. 2000; Brugge et al. 2003; Wallace et al. 2003; Rojas-Bracho et al. 2004; Allen et al. 2004, 2008; Olson and Burke 2006; Baxter et al. 2007; Brown et al. 2009; Hart et al. 2011; Ward et al. 2011; Noonan et al. 2012; Paulin et al. 2013; McNamara et al. 2013; Semmens et al. 2015; Fleisch et al. 2020; Zhao et al. 2020; Hadeed et al. 2021) and five in Canada (Allen et al. 2009; MacNeill et al. 2014; Wheeler et al. 2014; Jeong et al. 2019; Mendell et al. 2022). Included studies were published between 1997 and 2021 with 32 of 49 published since 2011 (Fig. 2).

The two most common types of measurement methods used to quantity PM_2.5_ concentrations were utilised in equal proportion across the studies; optical and gravimetric devices were each employed in 24 studies, with one study using both optical and gravimetric methods. Static sampling was adopted in 37 studies, four placed devices on participants and eight studies used both static and personal placements. Table 1 provides details of placement methods within each type of device.

**Table 1:** Placement methods within each type of device

Type of device	Device placement	Number of studies
Gravimetric	Both static and personal	6
	Personal	3
	Static	15
Optical	Both static and personal	1
	Personal	1
	Static	22
Both gravimetric and optical	Both static and personal	1

**Fig. 3:**
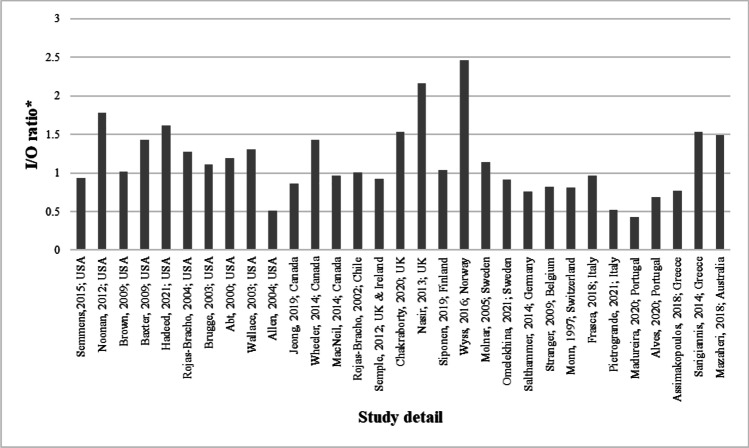
Calculated I/O ratios of included studies

Out of 49 studies, 40 reported methods in measuring concurrent outdoor PM_2.5_ concentrations. Data on indoor and outdoor PM_2.5_ concentrations was available for 31of these studies (Supplementary Information 3) and was extracted. Indoor-to-outdoor (I/O) ratios for each of the studies with complete extractable datasets are presented in Fig. 3. Values > 1 indicate a higher indoor PM_2.5_ concentration than that measured outdoors.

*I/O ratios are calculated based on measures of central tendency provided in individual original articles.

Of all included studies, 32 were rated as having a low risk of bias for their exposure assessment methods, 15 studies had medium risk, and only two were assessed as having high risk of bias. Most studies that were rated as medium risk were so, due to short sampling durations that would be insufficient in capturing behavioural variabilities; in this systematic review, insufficient sampling period was defined as being ≤ 72 h. The remaining medium risk studies failed to specify the location of sampling device placement, potentially resulting in measurement errors within individual included studies. Studies deemed as being at high risk of bias failed on both sampling duration and specificity of device placement.

### Sources of exposure

Although many studies investigated exposure sources other than those listed in Supplementary Information 1, only original studies that reported concentration in μg/m^3^ were included in this systematic review. Studies that did not report actual measurements relating to a particular source, but instead provided general values of static or personal PM_2.5_ concentrations, are included in the analysis as having “no specific source”.

#### Woodstoves

**Fig. 4:**
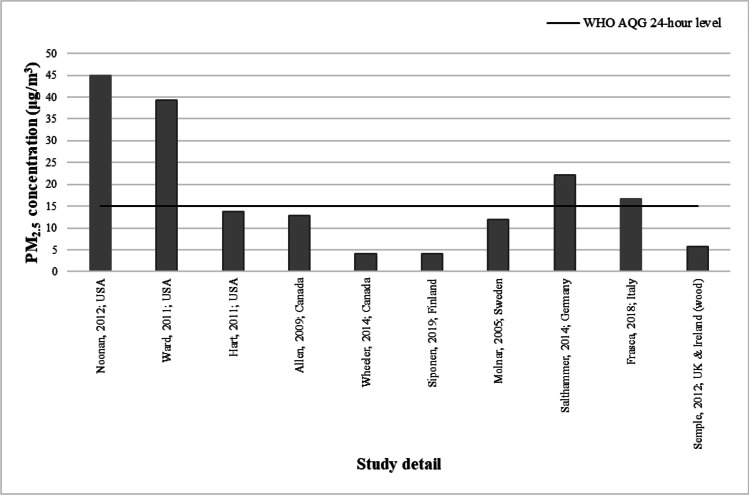
Studies that reported woodstove related PM_2.5_ concentration in a 24-h period

Indoor PM_2.5_ generated from woodstoves was measured in a total of 15 studies. The reported PM_2.5_ concentrations varied widely between studies, with 24-h mean or median ranging from as high as 45.0 μg/m^3^ (Noonan et al. 2012) down to 4.1 μg/m^3^ (Siponen et al. 2019). Figure 4 illustrates the varied concentration in the ten studies that report PM_2.5_ in woodstove using homes in a 24-h period and how they compare with the WHO AQG level. Not all studies reported their PM_2.5_ concentrations over 24 h. Two studies reported 48-h average PM_2.5_ concentrations of 28.8 (Semmens et al. 2015) and 32.3 (McNamara et al. 2013) μg/m^3^. A further two gave PM_2.5_ concentrations as hourly means of 12.2 (Chakraborty et al. 2020) and 26.4 (Wyss et al. 2016) μg/m^3^. Only one study (Fleisch et al. 2020) reported a weekly median PM_2.5_ value as being 6.65 μg/m^3^.

#### Solid fuel burning

Six studies investigated PM_2.5_ concentrations associated with solid fuel appliances other than just wood combustion. Two studies examined biomass-burning fireplaces, with one reported the daily mean being 31.1 μg/m^3^ (Marques and Pitarma 2020), and the other estimated the 24-h mean PM_2.5_ concentration at 50 μg/m^3^ during a cold period whilst fireplaces were operating (Sarigiannis et al. 2014). One study investigated two types of solid fuel combustion, coal and peat burning, with 24-h mean PM_2.5_ concentrations measured at 7.4 and 10.9 μg/m^3^, respectively (Semple et al. 2012). Coal/wood burning stoves were examined by two studies; one reported the average PM_2.5_ concentration in August as being 22.9 μg/m^3^ and in December as 15.0 μg/m^3^ (Paulin et al. 2013), whilst the other gave mean personal exposure to PM_2.5_ when coal/wood stoves were in operation as 48.2 μg/m^3^ (Jedrychowski et al. 2006). The 24-h mean PM_2.5_ concentration associated with solid fuel burning in general was reported by one study, giving 12.5 μg/m^3^ in the non-heating season and 33.9 μg/m^3^ during the heating season (Hadeed et al. 2021).

#### Cooking

A total of 16 studies examined PM_2.5_ concentrations related to cooking. Like the previous two sources of exposure, there is a great deal of variation between studies in terms of time periods in which the measurements were reported and in the concentration values themselves. Three studies reported the peak PM_2.5_ concentration during cooking. Omelekhina et al. (2022) reported a single peak value as high as 3050 μg/m^3^, whereas the other two studies provided averaged peak values. Zhao’s et al. demonstrated 5-min median peak PM_2.5_ concentration of 37 μg/m^3^ (Zhao et al. 2020), whilst Noonan’s study reported a median peak value an order of magnitude higher at 305 μg/m^3^ (Noonan et al. 2012). Four studies reported their PM_2.5_ concentrations in 24-h periods, with the values from the three studies (Semple et al. 2012; Siponen et al. 2019; Pietrogrande et al. 2021) conducted in Europe being relatively similar to one another, ranging from 3.1 to 18.7 μg/m^3^, whilst a 24-h mean value of 54.7 μg/m^3^ reported from a study in Kuwait is significantly higher (Yassin et al. 2012). One study reported its PM_2.5_ concentration as an hourly mean (Wyss et al. 2016), and another two studies as 48-h means (Wigzell et al. 2000; Jedrychowski et al. 2006). The remaining six studies (Olson and Burke 2006; Baxter et al. 2007; O’Leary et al. 2018; Mazaheri et al. 2018; Alves et al. 2020; Shehab et al. 2021) (Table 2) reported their cooking related PM_2.5_ concentrations over various sampling durations, thus making them difficult to group and directly compare.

**Table 2:** Studies examining cooking related PM_2.5_ that have not been described in main text

	Sampling duration	Central tendency	Main findings (μg/m^3^)	
Shehab et al. 2021	Four days	Mean	24.7–50.0	
O’Leary et al. 2018	Two weeks	Mean	Week 1	26.8–226
			Week 2	20.7–308
Alves et al. 2020	Two homes for 48 h, two for 72 h	Mean	14–30	
Mazaheri et al. 2018	One week	Mean	Weekdays	6.47–9.49
			Weekend	6.10–13.0
Baxter et al. 2007	Three to four days in two seasons	Mean	6.77–74.9	
Olson and Burke 2006	Seven days in each of the four seasons	Mean	42–377	

#### Candle and incense

Five studies characterised indoor PM_2.5_ associated with the use of candles and with a further one study investigating the burning of incense. The burning of incense was reported to increase indoor PM_2.5_ concentration by an average of 6 μg/m^3^ (Wallace et al. 2003). Two studies reported PM_2.5_ concentrations of 70 (Noonan et al. 2012) and 36 (Assimakopoulos et al. 2018) μg/m^3^ during candle burning, while the remaining three reported values over various time frame. For example, a mean hourly concentration of 20.3 μg/m^3^ was reported by Wyss et al. (2016); Jedrychowski et al. measured the 48-h mean personal exposure to PM_2.5_ during the burning of candles as being 45.6 μg/m^3^ (Jedrychowski et al. 2006); and the indoor daily median concentration in Siponen’s study was 4.2 μg/m^3^ (Siponen et al. 2019).

#### Cleaning

Two studies examined PM_2.5_ emission associated with household cleaning. One study reported the median peak PM_2.5_ during cleaning was 28 μg/m^3^ (Noonan et al. 2012), whereas the other found house cleaning activities led to a daily median indoor PM_2.5_ concentration of 4.5 μg/m^3^ (Siponen et al. 2019).

#### Humidifier

Only one study characterised PM_2.5_ associated with the use of a humidifier in a real-life setting; this is perhaps due to humidifiers not being common household items. Nevertheless, the use of a humidifier was shown to lead to an approximate five-fold increase when compared to ambient PM_2.5_ concentrations. From Brown’s study, the mean exposure was calculated to be 49.5 and 59.0 μg/m^3^ in winter and summer, respectively (Brown et al. 2009).

#### No specific source

As previously mentioned, not all studies related indoor PM_2.5_ concentrations to a specific emission source as 15 of the 49 studies measured general indoor PM_2.5_ levels at home. Despite the generality of these studies, they also show considerable variation in measurement and reporting methods. Abt used 12-h mean PM_2.5_ concentration across homes, reporting a value of 13.9 μg/m^3^ (Abt et al. 2000), whilst both Allen (Allen et al. 2004) and Jeong (Jeong et al. 2019) gave hourly mean concentrations between 5.9 to 8.7 μg/m^3^. Five studies reported means or medians over 24-h periods. Two of these studies had very similar values, with MacNeil reporting 6.78 μg/m^3^ in winter and 10.10 μg/m^3^ in summer (MacNeill et al. 2014), whilst Nasir’s saw PM_2.5_ concentrations of 6 and 9 μg/m^3^ in respective seasons (Nasir and Colbeck 2013). Stranger and Ohura also reported similar results, with Stranger reporting a mean PM_2.5_ concentration of 29.5 μg/m^3^ (Stranger et al. 2009), whilst Ohura found mean personal exposure in a living room during winter to be 35.3 μg/m^3^ and 16.5 μg/m^3^ in summer (Ohura et al. 2005). The one study conducted in Chile found 24-h mean personal exposure to be 69.5 μg/m^3^ and indoor static measurement to be 68.5 μg/m^3^ (Rojas-Bracho et al. 2002), which are significantly higher than the four studies conducted in Europe and North America. The remaining seven studies (Monn et al. 1997; Brugge et al. 2003; Rojas-Bracho et al. 2004; Allen et al. 2008; Karottki et al. 2014; Madureira et al. 2020; Mendell et al. 2022) investigating residential PM_2.5_ concentrations, not related to a particular source, all used a variety of measurement methods and reported their findings over time periods specified in the original articles (Table 3).

**Table 3:** Studies examining non-source specific PM_2.5_ concentrations that have not been described in main text

	Sampling duration	Central tendency	Main findings (μg/m^3^)		
Karottki et al. 2014	45 h	Median	11.8		
Madureira et al. 2020	48 h	Mean	31		
Monn et al. 1997	48 to 72 h	Mean	18.3–26.0		
Rojas-Bracho et al. 2004	One 6-day period in winter, one or two 2-day period(s) in summer	Mean		Winter	Summer
			Personal	2.6–128.0	0.6–68.9
			Indoor	3.5–73.2	1.6–52.1
Brugge et al. 2003	Six 24-h periods for 7 homes, three 24-h periods for 2 homes	Mean	12.3		
Allen et al. 2008	Ten days	Mean	5–19		
Mendell et al. 2022	Four 7-day periods	Median	2.7		

## Discussion

This review identifies only 49 studies, spanning a period of nearly 25 years, that have measured and quantified concentrations of PM_2.5_ in non-smoking homes in HICs. Many countries appear to have no published data on typical household concentrations. In the small number of studies identified, there is considerable variability that is often difficult to interpret due to the lack of qualitative or contextual data on the sources and individual behaviours of household residents. The burning of wood and other solid fuels, cooking, the burning of candles and incense, house cleaning and the use of humidifiers have all been studied to varying extent and shown to lead to household indoor PM_2.5_ concentrations in HICs that exceed the WHO AQG. Despite the many differences in study design and methodology making direct comparison between studies and the extraction of meaningful conclusions difficult, wood/solid fuel burning appliances appear to be the most likely to produce high concentrations of PM_2.5_ with almost half of the included studies concerning this source reporting values above the WHO AQG 24-h level. Very few studies concerning other sources of exposure report such high PM_2.5_ concentrations, although there are examples for each source that stand out as having the ability to generate high concentrations.

Although this review has identified that household sources within HICs can lead to indoor PM_2.5_ concentrations that exceed the WHO AQG; however, they tend to be much lower than those reported in LMICs. For example, the mean indoor PM_2.5_ concentrations in kitchens with traditional biomass or solid fuel burning stoves in LMICs can be between 530 and 990 μg/m^3^ (Pope et al. 2017), whereas in this review, the highest reported concentrations associated with similar sources are in the region of 50 μg/m^3^ in a 24-h period. This echoes similar findings in Lim’s review that concludes the personal exposure to PM_2.5_ in HICs is much lower than countries in other classifications by income levels (Lim et al. 2022). The difference in indoor PM_2.5_ concentrations between smoking and non-smoking homes in HICs is another avenue for comparison. From the included studies within this review where samples were obtained from both smoking and non-smoking homes,^1^ smoking, either from active smoking or second-hand smoke, led to indoor PM_2.5_ concentrations to increase by 5.7 to 37 μg/m^3^. It is important to be mindful of these values when comparing data and that there are many factors to consider when drawing conclusions from these results.

It is apparent from the review of literature that there are limited data on household indoor PM_2.5_ related to non-tobacco sources within HICs. These studies in HICs only started to emerge in the late 1990s with just two to three publications per year thereafter, culminating in a total of 49 studies. Despite the inherent difficulties of carrying out exposure assessment studies in LMICS, there is considerably more research in these settings. Due to the focus on indoor combustion in homes within LMICs, a systematic review conducted in 2017 identified 55 studies in LMICs that characterised indoor PM_2.5_ associated with the use of cookstoves (Quansah et al. 2017).

Within the literature on PM_2.5_ concentrations in homes within HICs, biomass and solid fuel burning for heating, followed by cooking fume, are the focus in most of the identified studies, whilst only a handful of studies investigated PM_2.5_ generated from house cleaning, the burning of candles and incense, and other PM-generating activities. There is also a geographical skew in the location of conducted studies, with the majority of studies carried out in North America and Europe. Other HICs, especially those in Asia, the Middle East, Oceania and South America, are seldom mentioned, creating significant gaps within the literature.

Within the limited literature on the indoor PM_2.5_ generated from household sources, there are two predominant methods utilised in the quantification of PM_2.5_; these are optical and gravimetric. Despite their widespread use within the field, there remain considerable differences, not only between measurement methods, but also within the two groups of devices, with variation arising between different models and brands based on the same measurement technology (Lanki et al. 2002; Wallace et al. 2011). At present, there appears to be no recognised standard procedure or calibration technique to correct for many of these differences. This problem in measurement is further complicated by the implementation of the measurement technique by researchers in different studies. Some studies use a static placement of the sampling device, whilst others adopt a personal device which yields PM_2.5_ concentrations as experienced by occupants within the study households (Adgate et al. 2003). This variability in measurement methods makes direct comparison across studies difficult. In addition to this challenge, results are reported using a wide range of averaging times and various measures of central tendency. For instance, studies that only report PM_2.5_ concentrations during the activity may produce exceptionally high values and thus not reflect a 24-h period rendering them incomparable against the WHO AQG 24-h level. As described earlier, the majority of studies were rated as having medium or high risk of bias due to exposure measurement methods failing to sample for more than 72 h. However, even with longer sampling periods, many behavioural variabilities may not be captured, making it difficult to estimate an annual average exposure, another WHO AQG metric. These factors highlight that without a standardised approach to the measurement and reporting of household PM_2.5_ concentrations, any meaningful comparison of data between studies is not only difficult but may also lack any validity. This closely echoes the conclusions and findings of another systematic review by Younger et al. (2022).

There would also appear to be a great degree of variability in the measured PM_2.5_ concentrations from the same source across and within studies, although, as just discussed, it is perhaps difficult to distinguish true variation in a source of exposure from the variation and uncertainty of the measurement device and method. Another consideration that may greatly impact measured values is contextual outdoor PM_2.5_ concentration. This significantly differs both temporally and spatially and will influence indoor PM_2.5_ concentration during the sampling, depending on house location and time of day and season (Cyrys et al. 2004; Chen and Zhao 2011). Among the studies herein collated, 31 of the 49 made reference to and had extractable outdoor PM_2.5_ measurements from either central monitoring sites or directly outside of participating homes. It is clear that outdoor concentrations are a consideration among researchers in the field. However, the overwhelming majority fail to report metrics such as building characteristics, ventilation and air exchange rate, among other structural and meteorological factors that would be required to comment on the effect that outdoor PM_2.5_ infiltration has on indoor measurements.

Further research is clearly required to build a more comprehensive picture of the exposure to indoor PM_2.5_ in homes within HICs. The contribution to this understanding, however, must be conducted and presented in a way that allows for ease of direct comparison between individual studies, such that meaningful conclusions may be drawn. Thus, there is an obvious need for standardised methods in both the measurement and reporting of indoor PM_2.5_ concentrations in this field of research. Such standardisation would perhaps be analogous to that called for in occupational exposure to hazardous substances (National Institute for Occupational Safety and Health 2002; Kromhout 2002). Parameters such as sources of exposure, times, locations and households would all need to be considered in such a standardised framework. Researchers should ensure that sampling devices, whether they be based on optical and gravimetric technologies, produce accurate, reliable and comparable values. This may be achieved by calibrating optical instruments by co-locating with reference gravimetric samplers. Values from optical instruments can then be reported after adjustment with these gravimetrically-derived calibrations (Wang et al. 2016; Vogt et al. 2021). Defining and standardising a minimum sampling duration that is representative of a household’s activity is another consideration that would greatly improve the validity of intra- and inter-study comparison. This data should then be reported in a standardised time weighted average and perhaps be consistent with that of the WHO AQG, which currently uses 24-h and annual average intervals for PM_2.5_ exposure. To allow for the comprehensive interpretation of data, as advocated in the field of occupational exposure, the collection and reporting of certain contextual information should be mandated. Examples of such information should include corresponding outdoor PM_2.5_ concentrations, building characteristics and ventilation conditions as a minimum.

An extrapolation that is pertinent to this review is the potential benefit of a low-cost PM_2.5_ monitor that provides instantaneous feedback. As already discussed, people in HICs spend a significant amount of time at home, and thus household sources that generate high levels of PM_2.5_ pose potential health risks to occupants that are unknowingly exposed for extended periods of time. Having easy and reliable access to real-time PM_2.5_ values may prompt residents to alter behaviours and limit their own exposures. This may include opening windows when cooking or minimising the use of candles. Such devices would provide the most benefit to individuals with existing respiratory conditions as a means of preventing the exacerbation of their illnesses which in turn may maintain or improve health, and reduce avoidable burden on the healthcare system.

### Strengths and weaknesses

There are several limitations to this systematic review. Firstly, the use of a single database for literature search may result in a very small number of studies being neglected from inclusion. PubMed, however, is likely to be the most comprehensive database for literature on indoor air quality in homes; thus, the omission should be minimum. Returned studies were single-screened based on their titles and abstracts by one researcher in the selection stage. Although 10% of these were independently assessed by a second researcher, it is still possible that relevant, but less explicitly so, studies were overlooked and not included. Their inclusion would not have been possible without screening the full-texts, an impracticable task for any systematic review of this kind. As only articles published in English were included, this systematic review would also have neglected a very small number of studies concerning indoor PM_2.5_ concentrations that have only been published in other languages. In addition to the limitation associated with the exclusion of potentially relevant studies, there are limitations associated with the extracted data itself. Due to the highly varied sample sizes and recruitment methods used across the included studies, the studies’ samples may not be representative of the target population, introducing bias and lowering the generalisability of the conclusions drawn from the review. The quality appraisal tool implemented in this systematic review to assess the risk of bias for the exposure assessment methods rather than the actual study designs themselves. It is therefore possible that this review includes studies with low external validity. The last noteworthy limitation pertains to the current lack of standardised methods for the measurement of PM_2.5_, with different studies using a variety of measurement devices and sampling durations, as discussed earlier. The potential observational errors in the included studies themselves can again negatively impact on the conclusions drawn.

## Conclusion

This systematic review collates existing studies concerning indoor PM_2.5_ concentrations associated with common household sources in HICs and reveals that these can, at times, generate PM_2.5_ concentrations inside homes that exceed the WHO AQG. The small number of studies identified in this review highlights the need for more research into concentrations of PM_2.5_ in homes within HICs. This review also provides insight into the current indoor PM_2.5_ measuring and reporting techniques which were found to vary greatly between studies. This high degree of variability in exposure assessments and the presentations of results suggests that more uniform and standardised methodologies are needed in future research. Most importantly, this systematic review highlights the need to promote public education around PM_2.5_ pollution in home settings and guide people to make more informed choices in lifestyles or behaviour. This should consequently reduce the health risks associated with exposure to high concentrations of PM_2.5_, and ultimately protect the health of people in HICs.

## Supplementary Information

Below is the link to the electronic supplementary material.

Supplementary file1 (DOCX 79 KB)

## Data Availability

Not applicable.

## Footnotes

1 This systematic review only included data from non-smoking homes; however, individual studies may have also reported data for smoking households.
