# Supplementary material for: Exposure to fine particulate matter (PM2.5) from non-tobacco sources in homes within high-income countries: a systematic review
Source: Air Qual Atmos Health. 2022 Nov 28;16(3):553–66. doi: 10.1007/s11869-022-01288-8 (PMC9703437; doi:10.1007/s11869-022-01288-8)
Supplement: Supplementary file 1 — Supplementary file1 (DOCX 79 KB) [file 11869_2022_1288_MOESM1_ESM.docx]

Supplementary information 1: Search strings used to retrieve studies on indoor PM_2.5_ concentrations in home settings within HICs

| Database | PubMed | |
| --- | --- | --- |
| Time period | November 2021 to January 2022 | |
| Strings | 1 | (wood stove*) AND (indoor) AND (particulate matter*) |
|  | 2 | (wood stove*) AND (indoor) AND (PM2.5) |
|  | 3 | (wood stove*) AND (indoor) AND (air quality) |
|  | 4 | (stove*) AND (particulate*) |
|  | 5 | (stove* OR kitchen*) AND (indoor OR household* OR residen*) AND (particulate* OR PM OR air quality) |
|  | 6 | (candle* OR incense*) AND (indoor OR household* OR residen*) AND (particulate* OR PM OR air quality) |
|  | 7 | (humidif*) AND (indoor OR household* OR residen* OR home*) AND (particulate* OR PM OR air quality) |
|  | 8 | (vacuum*) AND (indoor OR household* OR residen* OR home*) AND (particulate* OR PM OR air quality) NOT (china OR india OR peru OR nepal) |
|  | 9 | (mosquito coil* OR insect* repel*) AND (indoor OR household* OR residen* OR home*) AND (particulate* OR PM OR air quality) NOT (china OR india OR peru OR nepal OR ghana OR guatemala) |
|  | 10 | (peat*) AND (indoor OR household* OR residen* OR home*) AND (particulate* OR PM OR air quality) NOT (china OR india OR peru OR nepal OR ghana OR guatemala) |
|  | 11 | (combust* OR coal*) AND (indoor OR household* OR residen* OR home*) AND (particulate* OR PM OR PM2.5 OR air quality) NOT (chin* OR india* OR peru OR nepal* OR ghana OR guatemala OR chile* OR ethiopia OR africa* OR mexic*) NOT (tobacco*) |
|  | 12 | (wood stove* OR woodstove* OR stove*) AND (indoor OR household* OR residen* OR home*) AND (particulate* OR PM OR PM2.5 OR air quality) NOT (chin* OR india* OR peru OR nepal* OR ghana OR guatemala OR chile* OR ethiopia OR africa* OR mexic*) NOT (tobacco*) |
|  | 13 | (cook* OR cooking fume* OR kitchen* OR frying) AND (indoor OR household* OR residen* OR home*) AND (particulate* OR PM2.5 OR air quality) NOT (chin* OR india* OR peru OR nepal* OR ghana OR guatemala OR chile* OR ethiopia OR africa* OR mexic*) NOT (tobacco*) |
|  | 14 | (candle* OR incense*) AND (indoor OR household* OR residen* OR home*) AND (particulate* OR PM2.5 OR air quality) NOT (chin* OR india* OR peru OR nepal* OR ghana OR guatemala OR chile* OR ethiopia OR africa* OR mexic*) NOT (tobacco*) |
| Limits | None | |

Supplementary information 2: Main characteristics of the included studies

| Surname of first author;  Publication year;  Risk of bias (L/M/H) | Country;  Sampling period | Source of exposure | Type of measurement device;  Location of device;  Sampling duration;  Measured outdoor PM_2.5_? (Y/N) | Main extractable findings |
| --- | --- | --- | --- | --- |
| Semmens, 2015 | USA | Woodstove | Optical (static)  Living room  Two 48-hour periods  Yes | 48-hour mean indoor PM_2.5_ concentrations (μg/m^3^):   \| 1^st^ sampling \| 28.8 (s.d. 28.5) \| \| --- \| --- \| \| 2^nd^ sampling \| 29.1 (s.d. 30.1) \| |
| Low | Winter of 2011/2012 |  |  |  |
| Siponen, 2019 | Finland | Woodstove; Candle; Cooking; Cleaning | Gravimetric (static & personal)  Living room & on subjects  Four to six 22-hour periods  Yes | General daily mean PM_2.5_ concentrations (μg/m^3^):   \| Personal – 4.3 (s.d. 5.3) \| Indoor – 5.0 (s.d. 5.9) \| \| --- \| --- \|   Source specific daily median PM_2.5_ concentrations (μg/m^3^):   \| Woodstove \| Personal – 3.2 \| Indoor – 4.1 \| \| --- \| --- \| --- \| \| Candle \| Personal – 2.4 \| Indoor – 4.2 \| \| Cooking \| Personal – 3.1 \| Indoor – 4.4 \| \| Cleaning \| Personal – 3.6 \| Indoor – 4.5 \| |
| Low | Nov 2008 to May 2009 |  |  |  |
| McNamara, 2013 | USA | Woodstove | Optical (static)  Common living area  Two 48-hour periods  No | The average corrected PM_2.5_ concentration in the homes was 32.3 (s.d. 32.6) μg/m^3^. |
| Low | Nov 2010 to Mar 2011 &  Nov 2011 to Mar 2012 |  |  |  |
| Fleisch, 2020 | USA | Woodstove | Gravimetric (static)  Room where subjects spent most time in (exclude kitchen)  7 days  No | The weekly median (IQR) PM_2.5_ concentration was 6.65 (5.02) μg/m^3^.  In homes with vs. without a stove, PM_2.5_ was 20.6% higher (-10.6 to 62.6, 95% CI). |
| Low | 2014 to 2016 |  |  |  |
| Wyss, 2016 | Norway | Woodstove; Candle;  Cooking | Gravimetric (static)  Main living space  7 days  Yes | Hourly mean PM_2.5_ concentrations (μg/m^3^):   \| Woodstove \| Present – 26.4 \| Absent – 12.9 \| \| --- \| --- \| --- \| \| Candle \| Present – 20.3 \| Absent – 13.4 \| \| Frying food \| Present – 34.5 \| Absent – 13.1 \| |
| Low | Nov 2012 to Feb 2013 |  |  |  |
| Salthammer, 2014 | Germany | Wood-burning fireplace | Gravimetric (static)  ≈ 2 to 3m from the appliance  3 days  Yes | 24-hour mean PM_2.5_ concentrations (μg/m^3^):   \| Background \| 13.71 \| \| --- \| --- \| \| During firing \| 22.14 \| |
| Medium | Nov 2012 to Mar 2012 |  |  |  |
| Semple, 2012 | UK & Republic of Ireland | Coal burning; Peat burning; Wood burning; Cooking | Optical (static)  Main living area  24 hours  Yes | 24-hour mean PM_2.5_ concentrations (μg/m^3^):   \| Coal \| 7.4 (s.d. 2.0) \| \| --- \| --- \| \| Gas cooking \| 7.1 (s.d. 1.9) \| \| Peat \| 10.9 (s.d. 2.5) \| \| Wood \| 5.7 (s.d. 2.2) \| |
| Medium | Oct 2009 to Mar 2010 |  |  |  |
| Molnar, 2005 | Sweden | Woodstove | Gravimetric (static & personal)  Living room & on subjects  24 hours  Yes | 24-hour median PM_2.5_ concentrations (μg/m^3^):   \| Personal \| 18 (range 5.3 – 59) \| \| --- \| --- \| \| Indoor \| 12 (range 3.9 – 61) \| |
| Medium | Feb to Mar 2003 |  |  |  |
| Chakraborty, 2020 | UK | Woodstove | Optical (static)  Same room as the woodstove  4 weeks  Yes | The hourly mean PM_2.5_ concentration was 12.21 (s.d. 10.36) μg/m^3^. |
| Low | Jan to Apr 2020 |  |  |  |
| Allen, 2009 | Canada | Woodstove | Gravimetric (static)  Living room  Two consecutive 3-day periods  Yes | Median indoor PM_2.5_ concentrations (μg/m^3^):   \| Pre-change \| 12.8 \| \| --- \| --- \| \| Post-change \| 12.2 \| |
| Low | Nov 2007 to Apr 2008 |  |  |  |
| Noonan, 2012 | USA | Woodstove; Cleaning; Candle; Cooking | Optical (static)  Same room as the woodstove  Two 24-hour periods  Yes | 24-hour mean PM_2.5_ concentrations (μg/m^3^) for woodstove:   \| Pre-change \| 45.0 (s.d. 33.0) \| \| --- \| --- \| \| Post-change \| 21.0 (s.d. 19.2) \|   Median peak PM_2.5_ concentrations (range) (μg/m^3^):   \| Cleaning \| 28 (28 – 28) \| \| --- \| --- \| \| Candle \| 70 (70 – 70) \| \| Cooking \| 305 (43 – 1230) \| |
| Low | Winters of 2007/2008 & 2008/2009 |  |  |  |
| Frasca, 2018 | Italy | Woodstove | Gravimetric (static)  Same room as the woodstove  18 days  Yes | The average PM_2.5_ concentration measured indoor was very similar to the outdoor value – 16.7 μg/m^3^. |
| Low | 12^th^ to 29^th^ Mar 2014 |  |  |  |
| Wheeler, 2014 | Canada | Woodstove | Optical (static)  Living room  3 days  Yes | The 24-hour median PM_2.5_ concentration (μg/m^3^) at baseline was 4.15 (25^th^ percentile 3.35; 75^th^ percentile 6.31). |
| Medium | Dec 2009 to Apr 2010 |  |  |  |
| Hart, 2011 | USA | Woodstove | Optical (static)  1.95 meters away from stove  Ten 24-hour periods  No | Mean PM_2.5_ concentrations at baseline (μg/m^3^):   \| Home A \| 13.98 \| \| --- \| --- \| \| Home B \| 13.60 \| |
| Low | Spring 2008 |  |  |  |
| Jedrychowski, 2006 | Poland | Coal/wood stove; Candle; Cooking | Gravimetric (personal)  On subjects, or by bedside at night  48 hours  Yes | Mean PM_2.5_ concentrations (μg/m^3^):   \| Coal/wood \| 48.20 \| \| --- \| --- \| \| Candle \| 45.60 \| \| Gas cooking \| 36.94 \| |
| Medium | Nov 2000 to Mar 2003 |  |  |  |
| Ward, 2011 | USA | Woodstove | Optical (static)  Same room as the woodstove  24 to 48 hours  Yes | The 24-hour median PM_2.5_ concentration at baseline was 39.2 μg/m^3^. |
| Medium | Jan to Mar 2007 |  |  |  |
| Stranger, 2009 | Belgium | No specific source | Gravimetric (static)  Living room  24 hours  Yes | The 24-hour average PM_2.5_ concentration in non-smoking homes was 29.5 μg/m^3^ (range 10.9 – 74.3). |
| Medium | 2001 to 2002 |  |  |  |
| Brown, 2009 | USA | Ultrasonic humidifier | Gravimetric (static & personal)  Main activity room, and on subjects or nearby during stationary activity  7 days in winter and summer  Yes | Mean PM_2.5_ concentrations (μg/m^3^):   \| Winter \| Personal \| 12.0 (s.d. 6.0) \| \| --- \| --- \| --- \| \| Ambient \| 9.9 (s.d. 5.1) \| \| Summer \| Personal \| 10.0 (s.d. 6.2) \| \| Ambient \| 11.8 (s.d. 5.5) \|   For subjects (n=2) with humidifiers, personal PM_2.5_ exposures were approx. 5 times greater than corresponding ambient concentrations. |
| Low | Nov 1999 to Jan 2000 &  Jun to Jul 2000 |  |  |  |
| Baxter, 2006 | USA | Cooking | Gravimetric (static)  Main living space  3 to 4 days in two seasons  Yes | The mean PM_2.5_ concentration was 20.3 (s.d. 12.5) μg/m^3^.  Cooking for > 1 hr/day contributed to an increase of 5.71 μg/m^3^ in indoor PM_2.5_ concentration. |
| Low | 2003 to 2005 |  |  |  |
| Shehab, 2021 | UK | Cooking | Gravimetric (personal)  On subjects  4 consecutive days  Yes | In houses located away from busy roads, mean personal exposures to PM_2.5_ concentrations (μg/m^3^) during cooking were:   \| Gas stove \| 50.0 \| \| --- \| --- \| \| Electric stove \| 24.7 \| |
| Low | Dec 2014 to Mar 2016 |  |  |  |
| Olson, 2006 | USA | Cooking | Optical (static & personal)  Living room, and on subjects  7 consecutive days in each of the four seasons  Yes | Average PM_2.5_ concentration (μg/m^3^) from personal samplers:   \| All cooking events \| 188 \| \| --- \| --- \| \| Electric \| 230 \| \| Gas \| 133 \| |
| Low | Summer 2000 to Spring 2001 |  |  |  |
| Madureira, 2020 | Portugal | No specific source | Optical (static)  Living room  48 hours  Yes | The mean PM_2.5_ concentration in non-smoking homes was 31 μg/m^3^. |
| Medium | May 2018 to Feb 2019 |  |  |  |
| Marques, 2020 | Portugal | Biomass burning fireplace | Optical (static)  Kitchen  7 days  No | Daily mean PM_2.5_ concentration ranged from 15.35 to 69.37 μg/m^3^.  Mean PM_2.5_ concentration was 31.05 μg/m^3^ (s.d. 66.085). |
| Low | Mar 2020 |  |  |  |
| Zhao, 2020 | USA | Cooking | Optical (static)  Living room  7 days  Yes | Median of the peak 5-min PM_2.5_ concentration during emissions (μg/m^3^):   \| Houses \| 36 \| \| --- \| --- \| \| Apartments \| 37 \| |
| Low | 2016 to 2018 (houses)  2018 to 2019 (apartments) |  |  |  |
| Pietrogrande, 2021 | Italy | Cooking | Optical (static)  Kitchen and main bedroom  2 weeks in each sampling season  Yes | Daily mean PM_2.5_ concentration (s.d.) (μg/m^3^):   \|  \| Winter \| Spring \| Summer \| \| --- \| --- \| --- \| --- \| \| Bedroom 1 \| 15.4 (5.4) \| 10.2 (1.5) \| 18.7 (2.9) \| \| Bedroom 2 \| 14.9 (4.7) \| 11.1 (1.9) \| 14.1 (1.8) \| \| Kitchen 1 \| 15.4 (5.3) \| 10.3 (1.2) \| 11.1 (2.2) \| \| Kitchen 2 \| 14.6 (5.3) \| 8.6 (2.0) \| 9.1 (1.9) \| |
| Low | Jan to Feb 2020 May to Jun 2020 Jun to Jul 2020 |  |  |  |
| Wigzell, 2000 | UK | Cooking | Gravimetric (static)  Kitchen and living room  48 hours  No | 48-hour mean PM_2.5_ concentration in non-smoking homes (μg/m^3^):   \| Kitchen \| 11.8 \| \| --- \| --- \| \| Living room \| 10 \| |
| Medium | Jun – Jul 1999 |  |  |  |
| Nasir, 2013 | UK | No specific source | Optical (static)  Kitchen and living room  At least 14 days  Yes | 24-hour average PM_2.5_ concentration (μg/m^3^) in non-smoking homes:   \| Winter \| 6 (s.d. 2) \| \| --- \| --- \| \| Summer \| 9 (s.d. 4) \| |
| Low | 2004 to 2008 |  |  |  |
| Ohura, 2005 | Japan | No specific source | Gravimetric (static & personal)  Living room, bedroom and kitchen, and on subjects  24 hours  Yes | 24-hour mean personal PM_2.5_ concentration (μg/m^3^) in living rooms in non-smoking homes:   \| Winter \| 35.3 \| \| --- \| --- \| \| Summer \| 16.5 \| |
| Medium | Feb to Mar 2002 & Sept 2002 |  |  |  |
| Karottki, 2014 | Denmark | No specific source | Gravimetric (static)  Living room  45 hours  Yes | The median PM_2.5_ concentration was 11.8 μg/m^3^. |
| Medium | Oct 2022 to Feb 2012 |  |  |  |
| Paulin, 2013 | USA | Coal burning | Gravimetric (static)  Main living area  4 days over two seasons  No | Average indoor PM_2.5_ concentration in non-smoking homes (μg/m^3^):   \| Heating fuel \| PM_2.5_ in Aug \| PM_2.5_ in Dec \| \| --- \| --- \| --- \| \| Coal \| 22.9 \| 15.0 \| \| Propane \| 26.4 \| 34.3 \| \| Electric \| 10.0 \| 7.0 \| \| Electric \| 11.7 \| 7.7 \| \| Electric \| 10.4 \| 12.8 \| |
| Low | Jul to Dec 2011 |  |  |  |
| Sarigiannis, 2014 | Greece | Biomass burning fireplace | Gravimetric (static)  Location not reported  24 hours  Yes | (The following values are estimates from Fig.6 in the original article. Exact values are not provided in either the article or supplementary information)  Mean measured indoor PM_2.5_ concentration (μg/m^3^):   \| Warm period \| --- \| 25 \| \| --- \| --- \| --- \| \| Cold period \| Without fireplace \| 40 \| \|  \| With fireplace \| 50 \| |
| High | Oct 2012 to Apr 2013 |  |  |  |
| O’Leary, 2018 | UK | Cooking | Optical (static)  Kitchen  2 weeks  No | Mean PM_2.5_ concentration (s.d.) for the whole sampling duration (μg/m^3^):   \| House \| Week 1 \| Week 2 \| \| --- \| --- \| --- \| \| A \| 70.6 (400.1) \| 72.8 (248.9) \| \| B \| 36.9 (79.7) \| 20.7 (96.8) \| \| C \| 34.7 (81.7) \| 40.5 (178.0) \| \| D \| 226.3 (847.9) \| 307.9 (1079) \| \| E \| 26.8 (147.5) \| 28.4 (193.6) \| |
| Low | Oct 2016 to Apr 2018 |  |  |  |
| Alves, 2020 | Portugal | Cooking | Gravimetric (static)  Kitchen  Two homes for 48 hours, two for 72 hours  Yes | Mean PM_2.5_ concentration by fuel type (μg/m^3^):   \| Gas \| 20.6 (s.d. 10.9) \| \| --- \| --- \| \| Electricity \| 17.8 (s.d. 12.2) \|   Mean PM_2.5_ concentration in kitchen (μg/m^3^):   \| House 1 \| 13.8 \| \| --- \| --- \| \| House 2 \| 30.2 \| \| House 3 \| 16 (estimate from Fig.1) \| \| House 4 \| 22 (estimate from Fig.1) \| |
| Medium | Oct to Nov 2017 |  |  |  |
| Hadeed, 2021 | USA | Solid fuel burning | Optical (static)  Living room  Two 24-hour periods  Yes | 24-hour mean indoor PM_2.5_ concentration in homes without smoking or incense (μg/m^3^):   \| Non-heating season \| 12.5 (s.d. 15.3) \| \| --- \| --- \| \| Heating season \| 33.9 (s.d. 43.6) \| |
| Low | Not specified |  |  |  |
| Yassin, 2012 | Kuwait | Cooking | Optical (static)  Kitchen  24 hours  No | 24-hour mean PM_2.5_ concentration in non-smoking kitchens (μg/m^3^):   \| House 1 \| 46.2 \| \| --- \| --- \| \| House 3 \| 59.4 \| \| House 4 \| 58.56 \|   The mean indoor PM_2.5_ concentration was 37.6 μg/m^3^ in non-smoking homes. |
| Medium | Summer |  |  |  |
| Mazaheri, 2018 | Australia | Cooking | Optical (personal)  On subjects  1 week  Yes | Mean PM_2.5_ concentration (μg/m^3^):   \| Weekday \| Home-eating-cooking \| 8.51 \| \| --- \| --- \| --- \| \| Home-indoors \| 8.35 \| \| Weekend \| Home-eating-cooking \| 13.02 \| \| Home-indoors \| 7.47 \| |
| Low | May to Oct 2016 |  |  |  |
| Assimakopoulos, 2018 | Greece | Candle | Optical (static)  Apartment B – living room and balcony  Apartment D – living room  17 days  Yes | Average indoor PM_2.5_ concentration (μg/m^3^):   \| Apartment B \| 21.6 (s.d. 4.16) \| \| --- \| --- \| \| Apartment D \| 15.8 (s.d. 2.55) \|   Indoor PM_2.5_ concentration was 36 μg/m^3^ during candle burning (estimated from Fig.6). |
| Low | 10^th^ to 26^th^ Mar 2015 |  |  |  |
| Rojas-Bracho, 2004 | USA | No specific source | Gravimetric (static & personal)  Main activity room, and on subjects  One 6-day period in winter, one or two 6-day period(s) in summer  Yes | Mean PM_2.5_ concentration (s.d.) (μg/m^3^):   \|  \| Winter \| Summer \| \| --- \| --- \| --- \| \| Personal \| 21.6 (15.2) \| 21.5 (11.9) \| \| Indoor \| 17.2 (13.) \| 17.7 (14.9) \| |
| Low | Winters of 1996/1997 & Summer 1996 |  |  |  |
| Monn, 1997 | Switzerland | No specific source | Gravimetric (static)  Location not reported  48 to 72 hours  Yes | Mean indoor PM_2.5_ concentration (μg/m^3^):   \| Home A \| 18.3 \| \| --- \| --- \| \| Home C \| 26.0 \| |
| High | Winter 1996 |  |  |  |
| Brugge, 2003 | USA | No specific source | Gravimetric (static)  General living area and child’s room  Six 24-hour periods for 7 homes; three 24-hour periods for 2 homes  Yes | The mean PM_2.5_ concentration in non-smoking homes was 12.3 μg/m^3^. |
| Low | Jan to Jun 2000 |  |  |  |
| Abt, 2000 | USA | NO specific source | Gravimetric (static)  Adjacent to areas of the home where most activities occurred One or two 6-day period(s)  Yes | 12-hour mean PM_2.5_ concentration was 13.9 (s.d. 15.2) μg/m^3^. |
| Low | Winter and summer of 1996 |  |  |  |
| Wallace, 2003 | USA | Incense | Optical (static)  Mainly in living room, alternative locations included child’s bedroom or dining room  Up to three 2-week periods  Yes | The mean indoor PM_2.5_ concentration in non-smoking homes was 17.8 μg/m^3^.  Use of incense led to an average increase of 6 μg/m^3^. |
| Low | Sampling period not specified |  |  |  |
| Jeong, 2019 | Canada | No specific source | Optical (static)  Living room or open concept kitchen/ living-dining room  5 to 7 days  Yes | The hourly mean PM_2.5_ concentration for all homes was 6 (s.d. 9) μg/m^3^. |
| Low | Jun 2014 to Dec 2017 |  |  |  |
| Omelekhina, 2021 | Sweden | Cooking | Optical (static)  Living room or hall in 5 homes, kitchen in 2 homes  Three 7-day periods  Yes | Peak PM_2.5_ concentration was 3048.5 μg/m^3^ during cooking.  Average indoor PM_2.5_ concentration was 8.6 (s.d. 5.8) μg/m^3^. |
| Low | Dec to Mar (year unknown) |  |  |  |
| Mendell, 2021 | Canada | No specific source | Optical (static)  Living room  Four 7-day periods  No | The median PM_2.5_ concentration in non-smoking homes was 2.7 μg/m^3^. |
| Low | 2015 and 2017, spring and autumn |  |  |  |
| Allen, 2004 | USA | No specific source | Gravimetric (static) & optical (personal)  Location not reported  First two years: twenty-six 10-day periods; final year: six 5-day periods  Yes | Hourly mean personal exposure to PM_2.5_ when indoor at home (μg/m^3^):   \| Healthy \| 7.3 (s.d. 2.6) \| \| --- \| --- \| \| CHD \| 6.9 (s.d. 2.5) \| \| COPD \| 5.9 (s.d. 2.5) \| \| Asthma \| 8.7 (s.d. 2.1) \| |
| Medium | Oct 1999 to Mar 2002 |  |  |  |
| MacNeil, 2014 | Canada | No specific source | Optical (static)  Family or living room  Seven 24-hour periods in summer and winter  Yes | Daily median indoor PM_2.5_ concentration (μg/m^3^):   \| Winter \| 6.78 (Q1 = 4.55, Q3 = 13.41) \| \| --- \| --- \| \| Summer \| 10.10 (Q1 = 6.71, Q3 =15.53) \| |
| Low | Jan to Apr & Jun to Sept in 2009 |  |  |  |
| Rojas-Bracho, 2002 | Chile | No specific source | Gravimetric (static & personal)  Main activity room excluding kitchen, and on subjects  24 hours  Yes | 24-hour mean PM_2.5_ concentration (μg/m^3^):   \| Personal \| 69.5 (s.d. 24.8) \| \| --- \| --- \| \| Indoor \| 68.5 (s.d. 23.2) \| |
| Medium | Winters of 1998/1999 |  |  |  |
| Allen, 2008 | USA | No specific source | Gravimetric (personal)  On subjects  10 days  Yes | Mean personal exposure to PM_2.5_ concentration was 12.73 (s.d. 7.52) μg/m^3^. |
| Low | Dec 2000 to May 2001 |  |  |  |

Supplementary information 3: Extracted data for the calculation of I/O ratios

| Study | Central tendency | Indoor PM_2.5_ (μg/m^3^) | Outdoor PM_2.5_ (μg/m^3^) | Ratio | Notes |
| --- | --- | --- | --- | --- | --- |
| Semmens, 2015 | Median | 14.9 | 23 | 0.648 | Based on different time of the day |
|  |  | 8.4 | 14.9 | 0.564 |  |
|  |  | 15.4 | 15.9 | 0.969 |  |
|  |  | 13.3 | 11.1 | 1.20 |  |
|  |  | 14.3 | 12.6 | 1.13 |  |
|  |  | 25.4 | 21.2 | 1.20 |  |
| Siponen, 2019 | Mean | 5 | 4.8 | 1.04 |  |
| McNamara, 2013 | - | - | - | - | Did not measure outdoor PM_2.5_ |
| Fleisch, 2020 | - | - | - | - | Did not measure outdoor PM_2.5_ |
| Wyss, 2016 | Mean | 26.4 | 11 | 2.4 | Woodstove |
|  |  | 20.3 | 11 | 1.85 | Candle |
|  |  | 34.5 | 11 | 3.14 | Cooking |
| Salthammer, 2014 | Mean | 13.71 | 23.86 | 0.575 | Background |
|  |  | 22.14 | 23.71 | 0.934 | Fireplace on |
| Semple, 2012 | Mean | 7.4 | 8.2 | 0.902 | Coal |
|  |  | 7.1 | 8.2 | 0.866 | Cooking |
|  |  | 10 | 8.2 | 1.22 | Peat |
|  |  | 5.7 | 8.2 | 0.695 | Wood |
| Molnar, 2005 | Median | 12 | 10 | 1.2 | Two types of devices used for outdoor PM_2.5_ |
|  |  | 12 | 11 | 1.09 |  |
| Chakraborty, 2020 | Mean | 12.21 | 7.99 | 1.53 |  |
| Allen, 2009 | - | - | - | - | Changes in I/O ratios already reported in original article |
| Noonan, 2012 | Mean | 45 | 25.3 | 1.78 |  |
| Frasca, 2018 | Mean | 16.7 | 17.4 | 0.960 |  |
| Wheeler, 2014 | Median | 4.15 | 2.9 | 1.43 |  |
| Hart, 2011 | - | - | - | - | Did not measure outdoor PM_2.5_ |
| Jedrychowski, 2006 | - | - | - | - | No extractable outdoor PM_2.5_ provided |
| Ward, 2011 | - | - | - | - | Outdoor value given as mean, but indoor value is median |
| Stranger, 2009 | Mean | 29.5 | 36 | 0.819 |  |
| Brown, 2009 | Mean | 12 | 9.9 | 1.21 | Winter |
|  |  | 10 | 11.8 | 0.847 | Summer |
| Baxter, 2006 | Mean | 20.3 | 14.2 | 1.43 |  |
| Shehab, 2021 | - | - | - | - | No extractable outdoor PM_2.5_ provided |
| Olson, 2006 | - | - | - | - | No extractable outdoor PM_2.5_ provided |
| Madureira, 2020 | Mean | 31 | 72 | 0.431 |  |
| Marques, 2020 | - | - | - | - | Did not measure outdoor PM_2.5_ |
| Zhao, 2020 | - | - | - | - | No extractable outdoor PM_2.5_ provided |
| Pietrogrande, 2021 | Mean | 15.4 | 43.3 | 0.356 | Winter |
|  |  | 14.6 | 47.6 | 0.307 |  |
|  |  | 10.3 | 7.4 | 1.39 | Spring |
|  |  | 8.6 | 9.6 | 0.896 |  |
|  |  | 11.1 | 10 | 1.11 | Summer |
|  |  | 9.1 | 15.4 | 0.591 |  |
| Wigzell, 2000 | - | - | - | - | Did not measure outdoor PM_2.5_ |
| Nasir, 2013 | Mean | 39 | 18 | 2.17 |  |
| Ohura, 2005 | - | - | - | - | No extractable outdoor PM_2.5_ provided |
| Karottki, 2014 | - | - | - | - | No extractable outdoor PM_2.5_ provided |
| Paulin, 2013 | - | - | - | - | Did not measure outdoor PM_2.5_ |
| Sarigiannis, 2014 | Mean | 25 | 19.4 | 1.29 | Warm weather |
|  |  | 50 | 29.5 | 1.69 | Cold weather |
| O’Leary, 2018 | - | - | - | - | Did not measure outdoor PM_2.5_ |
| Alves, 2020 | Mean | 13.8 | 18.3 | 0.754 | House 1 |
|  |  | 16 | 27.6 | 0.580 | House 3 |
|  |  | 22 | 29.5 | 0.746 | House 4 |
| Hadeed, 2021 | Mean | 14.6 | 9.3 | 1.57 | Non-heating season |
|  |  | 36.2 | 22.1 | 1.64 | Heating season |
| Yassin, 2012 | - | - | - | - | Did not measure outdoor PM_2.5_ |
| Mazaheri, 2018 | Median | 9.62 | 6.44 | 1.49 | Weekend values |
| Assimakopoulos, 2018 | Mean | 21.6 | 24.2 | 0.893 | Apartment B |
|  |  | 15.8 | 24.2 | 0.653 | Apartment D |
| Rojas-Bracho, 2004 | Mean | 17.2 | 10.9 | 1.58 | Winter |
|  |  | 17.7 | 16.4 | 1.08 | Summer |
| Monn, 1997 | Mean | 18.3 | 33.9 | 0.540 | Home A |
|  |  | 26 | 21 | 1.24 | Home C |
| Brugge, 2003 | Mean | 12.3 | 13.1 | 0.940 | Outdoor monitor 1 |
|  |  | 12.3 | 9 | 1.37 | Outdoor monitor 2 |
| Abt, 2000 | Mean | 13.9 | 11.7 | 1.19 |  |
| Wallace, 2003 | Mean | 17.8 | 13.6 | 1.31 |  |
| Jeong, 2019 | Mean | 6 | 7 | 0.857 |  |
| Omelekhina, 2021 | Mean | 8.6 | 9.4 | 0.915 |  |
| Mendell, 2021 | - | - | - | - | Did not measure outdoor PM_2.5_ |
| Allen, 2004 | Mean | 6.5 | 12.7 | 0.512 |  |
| MacNeil, 2014 | Median | 6.78 | 7.69 | 0.882 | Winter daily |
|  |  | 10.1 | 9.76 | 1.03 | Summer daily |
| Rojas-Bracho, 2002 | Mean | 68.5 | 68.1 | 1.01 |  |
| Allen, 2008 | - | - | - | - | No extractable indoor PM_2.5_ provided |
